# Artificial Intelligence Think Tank: a modern problem-solving framework

**DOI:** 10.3389/frai.2025.1603562

**Published:** 2025-08-14

**Authors:** Shahryar Sorooshian

**Affiliations:** Department of Business Administration, University of Gothenburg, Gothenburg, Sweden

**Keywords:** artificial intelligence, human expertise, problem-solving, decision-making, Generative AI

In today's world, when everything is changing quickly and new global concerns are emerging, lifelong learning and creative problem solving are more crucial than ever. Classical approaches such as the brainstorming, Delphi, Nominal Group Technique, focus groups, and the World Café are some of existing problem-solving supporting approaches, but they may not necessarily suit complicated and extended decision-making situations (Caudle et al., 2025; Raadschelders and Whetsell, 2018; Watkins et al., 2012). These approaches are consensus-based and hence rely on the availability of experts, time, and cognitive capacity, which limits their scalability and effectiveness in dynamic contexts. Their drawbacks become particularly pronounced in emerging sectors, where access to sufficient expertise is often constrained by high costs, time pressures, or simply a lack of established specialists (Palonen et al., 2014). The drawbacks of these classic methods, such as their reliance on the availability of experts, significant time necessities, and limited scalability, may cause decisions to be delayed, opportunities to be missed, and solutions to be lacking in resource-constrained situations.

To address the classic approaches' limitations, a growing trend has emerged involving the utilization of artificial intelligence (AI) to support human capabilities across various domains (Korteling et al., 2021). According to Fui-Hoon Nah et al. (2023), AI–human collaboration has emerged as a promising path forward in addressing these challenges and unlocking new possibilities for human development. The technologies improve data mining, data analysis, and even certain decision-making activities that were previously the domain of human specialists, which might be very valuable in the cyclic learning process and future-oriented problem-solving. As a result, in order to capitalize on these prospects, this paper suggests the AI Think Tank (AITT) framework as a novel and unique method to decision surrogate modeling that may complement and replace existing ways to lean and progress for decision making and problem-solving. Current versions of Generative AI technology can produce human-like conversations and are gaining popularity due to their ability to give tailored and context-sensitive replies (Dwivedi et al., 2023). By integrating AI systems such as ChatGPT (https://chatgpt.com/), Microsoft Copilot (https://copilot.microsoft.com), Depseek (https://www.deepseek.com/), or Gemini (https://gemini.google.com/app) into a knowledge-based decision-making framework. AITT ensures comprehensive coverage of the situation by including decision-makers with limited knowledge of the context, which is useful in emerging, complex, and multifaceted scenarios where human expertise is scarce or difficult to access due to cost or time constraints. It also is closely tied to the purpose of creating new learning opportunities and possibilities for human development. The AITT procedure, as Figure 1 shows, has the power to promote inventive thinking and broaden the boundaries of how humans learn, adapt, and prosper in an ever-changing environment, by promoting continuous skill acquisition, improving decision-making efficiency, and encouraging cooperation between AI and human judgment.

**Figure 1:**
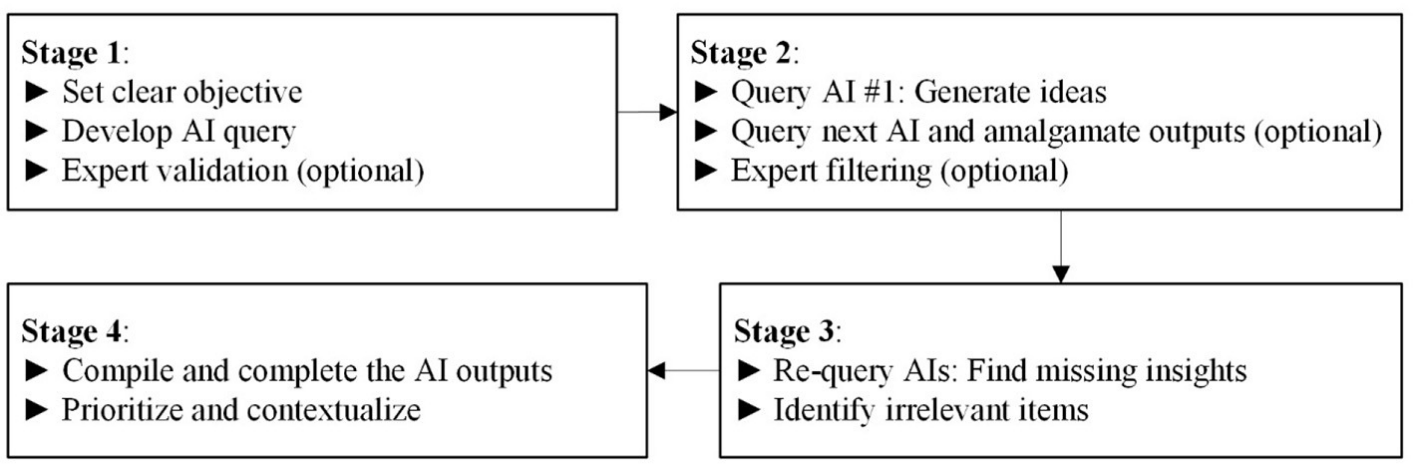
AITT flowchart.

The AITT's goal is to provide AI backing alongside incorporating characteristics of traditional decision-making methods. The proposed AITT framework has four main stages:

Stage 1: Decision-makers set clear problem-solving objective(s) and create a standardized query for making the necessary decision.

1. Decision-maker(s) define(s) a clear objective for the AITT use, specifying what problem/decision needs to be addressed.
2. Correspondingly, a comprehensive standardized prompt/query is developed by the decision maker(s) to ensure consistency and reliability in AI outputs. The prompt may include all necessary background information such as current trends, constraints, and goals.
3. If feasible, invite (a) field expert(s) to review and validate the prompt, mapping it to the problem/decision at hand.

Stage 2: Getting insights from AI. Although relying on a single AI chatbot remains possible, it is preferred that the standardized prompt be posed to multiple AI systems to ensure coverage of a broad spectrum of insights. Using different AIs ensures diversity in inputs, as each AI system (e.g., ChatGPT, Gemini) operates with unique data sources and methodologies, offering complementary perspectives.

1. Select an AI system and, according to the developed prompt, task it with generating ideas and needed problem-solving variables (such as success factors, barriers and challenges, motives, decision criteria, risks, etc.), or alternative solutions for a given problem.
2. When using more than one AI system, utilize the next AI with the following modified prompt: To address “[your prompt]”. I found “[paste the AI's output summary]”. Is there anything missing [enter what you're looking for, such as a fact, variable, solution, etc.]? Combine the AI outputs and repeat the process sequentially until all AI systems have been engaged.
3. It is beneficial, yet optional, that as a decision-maker (and if feasible, invite (a) field expert(s) to) review and filter out the AI outcomes based on problem/decision characteristics, for example analyzing the items relevance, importance, feasibility, etc.

Stage 3: Since the AITT process integrates insights from multiple AI sources, ensuring consistency in outputs is essential.

1. Use the utilized AIs from first to last, and the prompt will be: To “[your prompt]”. I searched for “[paste the AIs outputs' summary]”. Is anything missing [type in what you're looking for, such as a fact, variable, solution, etc.]? Continue to update the prompt and ask the AIs until no new answer is provided, or the answers are not relevant anymore.
2. This addition effort may also be beneficial to use the previously used AIs from first to last again, with the prompt “To [your prompt], I looked up [paste the AIs outputs' summary]. Is there anything irrelevant to [type in what you're looking for, like a fact, variable, solution, etc.]? If so, list it/hem and explain why it/they shouldn't be in the list”. As the decision-maker, and, if possible, invite (an) human expert(s), evaluate the AI-generated outputs to be considered or not.

Stage 4: The AI-generated responses are consolidated into a comprehensive list of ideas, variables, criteria, etc., in response to the developed prompt. Stage 4, thus, involves evaluating, organizing, and prioritizing the final set of AI-generated outputs. Human validation is essential at this stage to refine, contextualize, and ensure their practical applicability. Human validation and collaboration help refine and contextualize AI insights and ensures alignment with real-world applications.

1. As a decision-maker (and if feasible, invite (a) field expert(s) to) evaluate and validate the AI-generated outputs, if possible. They may also offer additional feedback and remarks.
2. As (a) decision-maker(s), use criteria like relevance and feasibility to evaluate outputs and authenticate insights according to the problem and/or decision context to minimize potential biases.

A simple case study was employed to illustrate the feasibility of the proposed AITT; it serves as a preliminary proof-of-concept. The aim of this case study was to demonstrate the potential application of the AITT framework. Hence, the four stages of the AITT were carried out in the explicitly defined order outlined lower. In this case study, we implement the AITT to identify its limitations.

1. We considered AITT validation in the scenario when there is no specific AITT expert available and the author is the sole proposer. Therefore, the AITT were utilized to provide feedback on the possible constraints of itself. Following the completion of the problem definition, the AITT method presentation was used to initiate this case study.
2. ChatGPT and Gemini were chosen for this case study due to their widespread popularity; just the two AI systems were used in the study to ensure a simple AITT implementation demonstration. A detailed description of the AITT framework was sent to both AI platforms, ChatGPT and Gemini, inquiring about potential limitations of the proposed AITT. The technique employed a standardized input for both AIs and offered the identical question to both: “What are the potential limitations of the AITT framework?”. Results were synthesized to create a comprehensive list, combining outputs from each AI system into a cohesive set of concepts.
3. Asking for more output, communication with AIs continued until no new answer, feasible, important, or reasonable output was provided. This stage contained the exclusion of items that received low agreement from the author or did not directly pertain to the AITT in relation to traditional problem-solving and decision-making methods.
4. A total of 21 concepts were incorporated in the aforementioned list, comprising 9 items from Gemini and 12 items from ChatGPT. Among these, 8 concepts showed either identical or extremely equivalent results when assessed by both ChatGPT and Gemini. Hence, a list of 13 was gathered and after reviewing the data summary, the authors, in their role as the decision-maker, concluded that some restrictions are more significant and should be explicitly communicated, though all listed items were valid.

By utilizing the AITT strategy, the decision-making scenario described above effectively collected and ranked ideas, indicating the potential for improved efficiency and comprehensiveness when compared with classic approaches, though more empirical validation remains required. This specific phase of strategic planning requires a substantial reduction in the time needed due to the automation of concept creation and analysis methods. The decision-maker determined that the developed concepts demonstrated proper logical consistency. The applied technique has shown its capacity to efficiently handle a wide range of inputs and adapt to different decision-making scenarios, without requiring the participation of a significant number of subject matter experts. Moreover, the employment of the AITT guaranteed the achievement of a thorough comprehension of important features and prerequisites for using the AITT approach, therefore offering an additional advantage.

The use of this specific case study confirmed AITT's applicability, demonstrating its capacity as a viable and efficient instrument for overcoming problem-solving challenges and reaching informed conclusions. Nevertheless, some potential limitations were identified. AITT results may be biased due to inconsistent AI performance, the risk of generating misplaced confidence, and occasionally, challenges in interpreting or explaining AI-generated reasoning clearly. These can be reduced, however, by employing cross-referencing techniques and human validation of AI outputs. AITT is unable to handle tacit knowledge; it also faces creativity and novelty limits because it works with documented information; and it is less able to fully consider emotional, cultural, intuitive understanding, common sense, and contextual nuance. To overcome these limitations, AITT users can follow best practices for more reliable and transparent use in practical applications. Human-in-the-loop supervision is still considered essential to adequately understand the results of AI and determine its usefulness and relevance. Users can evaluate AI responses with cross-validation to identify harmony or contradictions. They may need to perform iterative prompt refinement to improve output quality and monitor AI system upgrades for response coherence. Another challenge for AITT is complexity in prompt engineering and the risk of resulting information overload, yet this needs to be addressed by integrating human oversight and modification of prompts.

As discussed in this letter, AI can act as a think tank, assisting us with problem solving and decision-making. This letter proposed a supplementary, systematic, adaptable, and efficient AITT framework for modern problem-solving; this semi-automated idea generation saves time and resources, making AITT highly adaptable across diverse industries, contexts, and levels of complexity. However, more research in various sectors and situations is needed to determine the potential application and improvement of AITT. To advance this paradigm, real-world examples must be requested, investigations must be conducted, and more verification cooperation is required. Integration of AITT with other, traditional or modern, decision-making methods broadens the problem comprehension, even when expert human input is scarce or expensive, as future researchers can verify.

## References

[B1] Caudle K. E. Burlison J. D. Bourque M. S. Hoffman J. M. (2025). Research and scholarly methods: Consensus techniques. J. Am. Coll. Clin. Pharm. 8, 1–9. 10.1002/jac5.70052

[B2] Dwivedi Y. K. Kshetri N. Hughes L. Slade E. L. Jeyaraj A. Kar A. K.. (2023). Opinion paper: “so what if ChatGPT wrote it?” Multidisciplinary perspectives on opportunities, challenges and implications of generative conversational AI for research, practice and policy. Int. J. Inform. Manage. 71:102642. 10.1016/j.ijinfomgt.2023.102642

[B3] Fui-Hoon Nah F. Zheng R. Cai J. Siau K. Chen L. (2023). Generative AI and ChatGPT: Applications, Challenges, and AI-Human Collaboration. Oxfordshire: Taylor and Francis.

[B4] Korteling J. Van De Boer-Visschedijk G. C. Blankendaal R. A. Boonekamp R. C. Eikelboom A. R. (2021). Human-versus artificial intelligence. Front. Artif. Intell. 4:622364. 10.3389/frai.2021.622364 33981990 PMC8108480

[B5] Palonen T. Boshuizen H. P. Lehtinen E. (2014). “How expertise is created in emerging professional fields. Promoting, assessing, recognizing and certifying lifelong learning,” in International Perspectives and Practices. Berlin: Springer.

[B6] Raadschelders J. C. Whetsell T. A. (2018). Conceptualizing the landscape of decision making for complex problem solving. Int. J. Public Admin. 41, 1132–1144. 10.1080/01900692.2017.134794639426661

[B7] Watkins R. Meiers M. W. Visser Y. (2012). A Guide to Assessing Needs: Essential Tools for Collecting Information, Making Decisions, and Achieving Development Results. Washington, DC: World Bank Publications.

